# Association of *PICK1* and *BDNF* variations with increased risk of methamphetamine dependence among Iranian population: a case–control study

**DOI:** 10.1186/s12920-021-00873-7

**Published:** 2021-01-26

**Authors:** Amir Tajbakhsh, Maliheh Alimardani, Mahla Asghari, Soheila Abedini, Sohrab Saghafi Khadem, Abolfazl Nesaei Bajestani, Forough Alipoor, Maryam Alidoust, Amir Savardashtaki, Peyman Hashemian, Alireza Pasdar

**Affiliations:** 1 Pharmaceutical Sciences Research Center, Shiraz University of Medical Sciences, Shiraz, Iran; 2 Department of Medical Genetics and Molecular Medicine, Faculty of Medicine, Mashhad University of Medical Sciences, Mashhad, Iran; 3 Ibn-E-Sina and Dr Hejazi Psychiatry Hospital, University of Medical Sciences, Mashhad, Iran; 4 Department of Medical Genetics, Ayatollah Madani Hospital, Gonabad University of Medical Sciences, Gonabad, Iran; 5 Islamic Azad University Torbat-e Jam Branch, Torbat-e-Jam, Iran; 6 Epilepsy Research Center, Shiraz University of Medical Sciences, Shiraz, Iran; 7 Department of Medical Biotechnology, School of Advanced Medical Sciences and Technologies, Shiraz University of Medical Sciences, Shiraz, Iran; 8 Medical Genetics Research Centre, Mashhad University of Medical Sciences, Mashhad, Iran; 9 Psychiatry and Behavioral Sciences Research Center, Mashhad University of Medical Sciences, Mashhad, Iran; 10 Division of Applied Medicine, Faculty of Medicine, University of Aberdeen, Foresterhill, Aberdeen, UK

**Keywords:** Addiction, Drug abuse, Substance dependence, Substance use disorder (SUD), Dopamine pathway, Glutamate pathway, Variations, And polymorphisms

## Abstract

**Background:**

Genetic factors play an important role in susceptibility to methamphetamine dependency. In this line, protein that interact with C-kinase-1 (PICK1) and brain-derived neurotrophic factor (*BDNF*) genes are linked to methamphetamine dependence (substance use disorder). Thus, in a case–control study, we investigated the association between polymorphisms of *PICK1* and *BDNF* genes and methamphetamine dependence in an Iranian population.

**Methods:**

Total of 235 cases and 204 controls were recruited in a period between 2015 to 2018. The *PICK1-rs713729*, *-rs2076369* and *BDNF-rs6265* genotypes were determined via ARMS-PCR assay. Statistical analysis was performed, using SPSS 20.0, PHASE 2.1.1 program as well as SNP Analyzer 2.0.

**Results:**

In the present study, two polymorphisms including *PICK1-rs713729* (OR 1.38 (CI 1.08–1.52; *P*-_value_ 0.004) in multiplicative and dominant models, and *PICK1-rs2076369* (OR 1.31 (CI 1.10–1.56; *P*-_value_ 0.002) in multiplicative, dominant and co-dominant models were associated with the risk of methamphetamine abuse. Moreover, haplotype analysis showed a significant association of haplotype AG (OR 2.50 (CI 1.50–4.16; *P*-_value_ 0.0002) in dominant, recessive and co-dominant models, and haplotype TT (OR 0.67 (CI 0.50–0.91; *P*-_value_ 0.009) in dominant and co-dominant models with the risk of methamphetamine abuse. None of the polymorphisms in this study had a high level of linkage disequilibrium.

**Conclusion:**

Our findings indicate that the *PICK1* gene polymorphism might affect the risk of methamphetamine dependency in our population.

## Background

Methamphetamine is the most extensively abused illegal drug and this is a growing global problem [1–3]. Many changes occur in the human brain as a result of methamphetamine abuse [4]. Methamphetamine leads to the release of synaptic dopamine, which might be a reason for the increased addiction [4]. In this context, methamphetamine can have neurotoxic effects on dopaminergic neurons and also contribute to changes in another neurotransmitter system, particularly glutamate [5, 6]. Glutamate-mediated excitotoxicity is the major mechanism by which methamphetamine damages the central nervous system (CNS) [7, 8]. Moreover, the primary target of methamphetamine is the dopamine transporters, which remove dopamine from the extracellular space at the synapse and controls dopamine signals [9]. The function and surface availability of the dopamine transporter are regulated via different cellular mechanisms [10, 11]. Additionally, it was stated that the density of dopamine transporter is low in the caudate/putamen of methamphetamine abusers, suggesting that the long-term use of methamphetamine results in damage to dopaminergic neurons [12, 13].

Several factors are involved in methamphetamine abuse. In this line, genetic factors play an important role in susceptibility to the use of methamphetamine [1–3]. The dopamine transporter, polymorphisms have been shown to be a risk factor for prognosis of prolonged-type methamphetamine psychosis, such as a single nucleotide polymorphism (SNP) [14, 15]. Moreover, in a systematic review, Bousman et al*.* showed that several genes are involved in methamphetamine-related disorders. It has been shown that three genes including catechol-O-methyltransferase (*COMT*), gamma-aminobutyric acid type A receptor subunit alpha1 (*GABRA1*) and dopamine receptor D4 (*DRD4*), nine genes including, brain-derived neurotrophic factor (*BDNF*), arrestin beta 2 (*ARRB2*)*,* cytochrome P450 2D6 (*CYP2D6*), glutathione S-transferase mu 1 (*GSTM1*), glycine transporter-1 (*GLYT1*), glutathione S-transferase P1 (*GSTP1*), solute carrier family 22 member 3 (*SLC22A3*), prodynorphin (*PDYN*), and protein interacting with C kinase (*PICK1*), two genes including gamma-aminobutyric acid receptor subunit gamma-2 (*GABRG2*) and v-akt murine thymoma viral oncogene homolog 1 (*AKT1*) seem to be associated with methamphetamine abuse or dependence in Japanese and Han-Chinese populations [15]. It is indicated that the genetic epidemiology of methamphetamine abuse is very complex. There is an association between *PICK1* gene and drug addiction, such as methamphetamine abuse [16]. In this respect, the *PICK1* gene is mapped to chromosome *22q13.1* [17]. Interestingly, PICK1 interacts with dopamine transporter, which leads to the clustering of dopamine transporter on the cell surface and subsequently the improvement of dopamine transporter uptake activity [16, 18].

Furthermore, *BDNF-rs6265* is a functional SNP, which affects drug dependency [19]. *BDNF* gene is mapped to chromosome *11p*. It was found that *BDNF-rs6265* (Val66Met) SNP is linked to susceptibility to methamphetamine dependence in a Thai and Malaysian population [20–22]. In this respect, BDNF proteins are involved in the regulation of synaptic transmission as well as the process underlying substance use disorder (SUD) [23]. BDNF was shown to support the survival and protection of dopaminergic neurons following methamphetamine administration in mice [6]. The functional effects of SNPs, which are linked to SUD, are often unclear; hence, more investigations are required on different populations to define how these variants influence gene expression and function. In this case, worldwide participatory attempts are warranted to promote the accessibility of large population-based datasets/samples and to increase the feasibility of genetic association studies [15]. On the other hand, a systematic review by Alam-MehrJerdi et al*.* indicated that the state of methamphetamine abuse requires further research on the epidemiology and health-related implications in Iran [24]. Therefore, we aimed to investigate the association between *PICK1* and *BDNF* main SNPs and methamphetamine dependence (substance use disorder (SUD)) in a cohort of Iranian individuals.

## Methods

### The aim, design and setting of the study

This case–control study involved 235 cases (with methamphetamine dependence (SUD)) and 204 gender-matched controls (healthy individuals). In our study, SUD was recognized by the 11 criteria. The 11 criteria are divided into four categories of behavior, such as impaired control, social impairment, risky use and pharmacological indicators (tolerance and withdrawal) related to the substance use (Table 1) [25, 26].

**Table 1:** Criteria for substance use disorders (SUD)

Num	Categories of behaviour	Criteria for Substance Use Disorders (SUD)*
1	Impaired control	*Used larger amounts or longer*: Taking the drug in greater quantities or over prolonged periods of time
2		*Repeated attempts to control use and/or quit:* Wanting to cut or avoid using the substance, but they haven't been successful
3		*Much time spent using*: Spending a lot of time to get, using, or recover from substance using
4		*Craving*: Cravings and encourages the substance to be used
5	Social impairment	*Activities given up to use:* Not able to do what you can do at home, at work, or at school that you once liked because of substance use
6		*Social or interpersonal problems related to use*: Continuing to use, even though it creates issues in your relationships or conflicts with others
7		*Neglected major roles to use:* Giving up and refusing to perform significant social, occupational or recreational functions as a result of substance use
8	Risky use	*Hazardous use*: Using substances again and again, including though you or others are in danger
9		*Social or interpersonal problems related to use*: Continuing to use, even though you know that you have a physical or psychological condition which may have been triggered or exacerbated by the substance
10	Pharmacological indicators	*Tolerance*: Need more substance to have the effect you like
11		*Withdrawal*: Development of withdrawal symptoms and signs of withdrawal, which can be eased by taking more of the substance

*In the present study, substance use disorders (SUD) is recognized by these 11 criteria

The *PICK1-rs713729*, *PICK1-rs2076369* and *BDNF-rs6265* genotypes were analyzed by amplification refractory mutation system-polymerase chain reaction (ARMS-PCR) assay. Subsequently, statistical analysis was performed, using SPSS 20.0 (IBM Inc., Chicago, IL, USA), PHASE 2.1.1 program as well as SNP Analyzer 2.0.

### The characteristics of participants

#### Study population and clinical data

This study was performed in accordance with the Declaration of Helsinki (1964) and its subsequent amendments. Moreover, approval was obtained from the local Ethics committee of Mashhad University of Medical Sciences (IR.MUMS.fm.REC.1394.421), 439 blood samples were collected from 204 controls and 235 cases from Mashhad, Iran from 2015 to 2018. After explaining the study objectives, a written informed consent was obtained from all participants. A questionnaire was used to collect demographic and other essential information from all participants (Table 2). The selection procedure included confirmed urine test (addiction test) and also the availability of complete patient’s follow-up data. Moreover, healthy participants, individually matched on age, were recruited from the Health Examination Centre who were receiving routine medical examinations.

**Table 2:** Demographic and clinical characteristics of controls and methamphetamine dependence (SUD)

Variable		Controls	Methamphetamine dependence (SUD)	*P*-_value_^*^
Gender	Female	38.9%	35.7%	P < 0.001
	Male	68.1%	64.3%	
Age (SD)		31.96 (8.44 ± 0.58)	38.91 (9.11 ± 0.68)	P = 0.423
Marriage status	Married	56.7%	43.4%	P < 0.001
	Separated	-	11.6%	
	Widow	-	4.2%	
	Divorced	1.1%	20.6%	
	Single	42.1%	20.1%	

*Significant P-_value_ < 0.05. SD: Standard deviation

### Description of materials

#### Blood collection and DNA extraction

In order to extract the DNA, approximately 10 millilitres (ml) of peripheral blood was obtained from all participants and immediately subdivided into tubes containing sterile ethylene diamine tetra acetic acid (EDTA) [27]. DNA extraction from whole blood was extracted using salting-out technique. Then, the extracted DNA was quantified by the ratio of absorbance at 260 nm (nm) and 280 nm (A_*260*/280_) via BioTek™ Epoch™ Microplate Spectrophotometer (Winooski, VT, USA,) as well as via gel electrophoresis and finally stored at -20 °C until used.

#### Target single nucleotide polymorphisms (SNPs) determinations (Marker selection)

In this study, the SNPs were selected using available SNPs databases and published articles. Such articles examined intron and exon SNPs, which might alter the affinity of *PICK1-rs713729*, *PICK1-rs2076369* and *BDNF-rs6265* to methamphetamine dependence (SUD) (Table 3). Moreover, potential functional SNPs were included in order to meet the following criteria: minor allele frequency (MAF) > 0.05 (5%), heterozygosity > 0.15 (15%) and also validated SNPs in articles and databases. Furthermore, in order to inhibit redundancy in SNPs genotyping, SNPs that are not located in strong linkage disequilibrium (LD) were chosen.

**Table 3:** Characteristics of the investigated polymorphisms in this study

Rs number	Gene	Protein	Position	Exon/ Intron	Variant length	Allele	Function	Haplotype distance (bp)
rs713729	*PICK1*	Non-coding	22:38,059,462	Intron 3	1	T > A	Intron variant	8183
rs2076369	*PICK1*	Non-coding	22:38,067,645	Intron 4	1	T > G	Intron variant	
rs6265	*BDNF*	NP_001137277.1:p.Val66Met	11:27,658,369	Exon 4	1	C > T	Missense	-

PICK1: Protein that interact with C-kinase-1; BDNF: Brain-derived neurotrophic factor; Val66Met (also called rs6265): Met: Methionine and Val: Valine; METH: Methamphetamine; bp: Base pairs

#### Genotyping

To determine the genotype frequency of *PICK1-rs713729*, *PICK1-rs2076369* and *BDNF-rs6265* an ARMS-PCR method was used. Specific primers for PCR amplification were designed via web tools, such as Primer1 and also WASP (web-based allele-specific primer designing tool) [28].

PCR amplifications for *PICK1-rs713729, PICK1-rs2076369* and *BDNF-rs6265* were conducted in a 10–15 µl (μl) volume per reaction, containing 3 µl Taq 2 × master mix (Ampliqon, Germany), 10 µM of each primer and 100 nanogram (ng) DNA. Moreover, the specific primers used to detect *PICK1-rs713729, PICK1-rs2076369* and *BDNF-rs6265* SNPs are listed in Table 4. For *PICK1-rs2076369*, we also used betaine (Ampliqon, Germany) as an enhancer in PCR.

**Table 4:** Primer sequences used for genotyping in ARMS-PCR

SNPs	Primers	Sequences	Primer Length (bp)	PCR products (bp)
rs6265	FO	CTACAGTTCCACCAGGTGAGAAGAGTG	27	400
	RO	ATGGACATGTTTGCAGCATCTAGGTA	26	
	FI (c)	TGGTCCTCATCCAACAGCTCTTCTATaAC	29	253
	RI(t)	TTGGCTGACACTTTCGAACcCA	22	201
rs713729	FO	CTTTCTAGCGGAATCCCGACTGTG	24	407
	RO	CAGTGAAAAAGCAAACCAGGACACTG	26	
	FI(a)	CTTCTCATTCTTGAGGTCTGACCCACA	27	196
	RI(t)	AGGTGGTCAGAAAGCCCCTCAGA	23	265
rs2076369	FO	CATGTTGCCCAAGCTGGTCTCAAACTC	27	299
	RO	CTGGACACCCGTAACTGCTCTGACC	35	
	FI(g)	AGGAGTCTCAGTCCAGAACAGTCTTGACG	29	191
	RI(t)	CTCCACACCCTGAGCCCCTTCTCA	24	165

Expected product size in bps depending on the SNPs. FO: Forward outer primer; FI: Forward inner primer; RI: Reverse inner primer; RO: Reverse outer primer; bp: Base pairs; ARMS-PCR: Amplification refractory mutation system-polymerase chain reaction; SNP: Single nucleotide polymorphism

The ARMS-PCRs condition for each primer is as follows, Table 5**.** In general, initial denaturation at temperature 94 °C for five minutes, then 35 cycles including denaturation at 94 °C for 25 s, annealing at alternative °C for 25 s (based on each primer), an elongation at 72 °C for 30 s followed by 72 °C for seven minutes as the final elongation step (Table 5).

**Table 5:** The ARMS-PCRs condition for targeted SNPs

SNPs	Primers	First Denaturation		35 cycles						Last extension	
		Tm°C	Min	Denaturation		Annealing		Extension		Tm°C	Min
				Tm°C	Min	Tm°C	Min	Tm°C	Min		
rs6265	FO	94 °C	7 min	94 °C	30 s	61.5	25 s	72 °C	45 s	72 °C	7 min
	RO										
	FI (c)										
	RI(t)										
rs713729	FO					61	30 s		30 s		5 min
	RO										
	FI(a)										
	RI(t)										
rs2076369	FO					64	25 s		30 s		5 min
	RI(t)										
	FI(g)					67	30 s				
	RO										

FO: Forward outer primer; FI: Forward inner primer; RI: Reverse inner primer; RO: Reverse outer primer; TM: Temperature; Min: Minute; S: Seconds; °C: Centigrade; ARMS-PCR: Amplification refractory mutation system-polymerase chain reaction; SNP: Single nucleotide polymorphism

Absence or presence of mutant or normal PCR products were detected via gel electrophoresis in 3% agarose gel by ultraviolet (UV) trans illuminator (Gel Doc; U:Genius).

### Statistical analysis

A Hardy–Weinberg equilibrium (HWE) method was used to evaluate the differences in data for statistical significance. HWE assumption was investigated by the Pearson *χ*^*2*^ distribution with 1 degree of freedom. Allele and genotype frequencies were calculated, and the differences between groups were evaluated by Chi-squared tests. Then, the association between methamphetamine, risk factors and alleles/genotypes was evaluated by binary logistic regression, estimating Odds ratios (ORs) and also 95% confidence intervals (CIs). Three logistic regression models were used to analyse the SNPs, using different genetic models (additive, dominant, and recessive). For the analysis of SNP-SNP interactions, an adjusted logistic regression model was used to estimate the multiplicative interaction effect of the SNPs, located on the same haplotype. *P*-_value_ = 0 < 0.05 was considered to be statistically significant. SPSS 20.0 (Inc., Chicago, IL, USA), PHASE program as well as SNP Analyser 2 software were used for further statistical analysis [29].

### Haplotype analysis

Haplotypes were generated and assembled from the genotyped data by PHASE program, to reconstruct haplotypes, and SNP Analyzer 2 software [29, 30]. In the present study, *P*-_values_ of less than 0.05 were considered to be statistically significant. Moreover, Bonferroni correction was also used to account for multiple testing; thus, a two-tailed *P*-_value_ < 0.016 (_=_0.05/3 SNPs) was considered to be statistically significant in the present study.

## Results

### Identification of single nucleotide polymorphisms (SNPs) and association studies

There were no significant associations between *BDNF-rs6265* and the risk of methamphetamine dependence (SUD). On the contrary, a significant association was observed between two SNPs and the risk of methamphetamine abuse including *PICK1-rs713729* (OR 1.38 (CI 1.08–1.52; *P*-_value_ 0.004) in multiplicative and dominant models, and *PICK1-rs2076369* (OR 1.31 (CI 1.10–1.56; *P*-_value_ 0.002) in multiplicative, dominant and co-dominant models (Table 6). Moreover, haplotype analysis showed that specific haplotypes related to these SNPs were associated with methamphetamine dependence (SUD). In this line, analysis of *PICK1-rs713729* and *PICK1-rs2076369* haplotypes in our population showed that the haplotype AG (OR 2.50 (CI 1.50–4.16; *P*-_value_ 0.0002) in dominant, recessive and co-dominant models and haplotype TT (OR 0.67 (CI 0.50–0.91; *P*-_value_ 0.009) in dominant model and co-dominant model had a significant association with the risk of methamphetamine dependence SUD) (Table 7).

**Table 6:** Analysis based on different genetic models

SNP number	Gene	Position	Genetic models	OR	95%CI	*P*_-value_*	Bonferroni correction
							P_-value_
rs713729	*PICK1*	*22: 38,059,462*	Multiplicative	2.12	1.34–3.36	**0.001**	0.003
			Dominant	2.08	1.21–3.57	**0.007**	0.020
			Recessive	2.51	0.97–6.52	0.05	0.149
			Co-dominant	2.71	1.04–7.06	**0.02**	0.061
rs2076369	*PICK1*	*22: 38,067,645*	Multiplicative	0.68	0.05–0.91	**0.009**	0.028
			Dominant	0.51	0.34–0.76	**0.0008**	0.003
			Recessive	0.92	0.51–1.67	0.806	-
			Co-dominant	0.64	0.34–1.21	**0.003**	0.008
rs6265	*BDNF*	*11: 27,658,369*	Multiplicative	1.09	0.74–1.61	0.65	-
			Dominant	1.05	0.68–1.63	0.81	-
			Recessive	1.75	0.43–7.13	0.64	-
			Co-dominant	1.76	0.43–7.18	0.89	-

*PICK1*: Protein interacting with C-kinase-1; *BDNF*: Brain-derived neurotrophic factor; SNP: Single nucleotide polymorphism. OR: Odds ratio; CI: Confidence interval; *P*_*-*values_ were obtained from Chi-square tests; *Significant of *P*-_value_ < 0.05; The association between methamphetamine, risk factors and alleles/genotypes was evaluated by binary logistic regression, estimating ORs and also CIs. Note: Significant *P*_*-*value_ in bold; Bonferroni corrected *P*_-value_ is 0.016

**Table 7:** Haplotype analysis based on different genetic models

Genetic models	Haplotype	*P-*_value_^*^	OR	Lower CI	Higher CI
Multiplicative	H3	**0.0002**	2.5	1.502	4.161
	H2	**0.009**	0.678	0.505	0.91
	H1	0.742	1.047	0.794	1.382
	H4	0.745	0.84	0.292	2.416
Dominant	H2	**0.001**	0.532	0.358	0.79
	H3	**0.007**	2.123	1.217	3.704
	H1	0.735	0.924	0.582	1.466
	H4	0.762	0.838	0.266	2.643
Recessive	H3	**0.003**	11.569	1.499	89.293
	H1	0.436	1.175	0.782	1.766
	H4	0.555	0.842	0.052	13.549
	H2	0.651	0.867	0.466	1.611
Co-dominant	H3	**0.004**	12.331	1.595	95.316
	H2	**0.006**	0.627	0.326	1.206
	H1	0.571	1.044	0.620	1.757
	H4	0.840	0.838	0.052	13.492

H1: TG; H2: TT; H3: AT; H4: AG; OR: Odds ratio; CI: Confidence interval; *P*-_*values*_ were obtained from Chi-square tests. * Significant of *P*-_*value*_ < 0.05; Significant *P*-_*value*_ in bold

### Distribution of single nucleotide polymorphisms (SNPs)

The frequencies of the genotypes with high-quality genotype call were as follows: *PICK1-rs713729* “TT” 174 (77.67%), “AT” 33 (14.73%), and “AA” 17 (7.58%) among cases and “TT” 168 (87.95%), “AT” 17 (8.90%) and “AA” 6 (3.14%) among controls; *PICK1-rs2076369* “GG” 117 (52.23%), “GT” 80 (35.71%) and “TT” 27 (12.05%) among cases and 70 (36.08%), 100 (51.54%), 24 (12.37%) and among controls, respectively. Moreover, *BDNF-rs6265* genotype frequencies were: “CC” 161 (72.52%), “CT” 55 (24.77%) and “TT” 6 (2.70%) among cases and 142 (73.57%), 48 (24.87%) and 3 (1.55%) among controls, respectively. Table 7 shows the conferred risk by each haplotype. In this study, two haplotypes, AG and TT were significantly different between the normal and methamphetamine dependence (SUD) individuals. The prevalence of TT haplotype in the case group (27%) was lower than that of the normal individuals (34%), and the frequency of the AG haplotype in the methamphetamine dependence (SUD) individuals was higher (12%) than that (3%) of the normal individuals (Additional file 1: Table 1). We also examined whether the three SNPs were in LD. In this line, there was no strong LD between these SNPs (Additional file 2: Table 1).

## Discussion

The SUD is a persistent relapsing disorder with harmful consequences [31]. In this respect, the brain reward system such as mesocortical dopamine system is the common feature of this disorder [32, 33]. Furthermore, abnormal dopamine and glutamate systems are associated with the pathophysiology of SUD and dependency. However, the development of drug dependency is affected via factors, such as pharmacological effects on mental status, environmental and individual factors, such as genetics. In this respect, genetic factors are suggested to have a greater effect on drug dependency. It was shown that drugs abuse and addiction cause dopamine and opioid peptides to be released into the ventral striatum, which causes “high” sensation in abusers [33, 34].

The use of methamphetamine has become a serious health concern in Iranian methadone dependent patients [24, 35]. In Iran, it is used to improve sexual performance and promote physical energy due to its stimulating effects [36]. It is shown that methamphetamine abuse has surged from 3.9% amongst both genders in 2007 to 60.3% for men in 2014 and 89.5% for women in 2015–2016. Remarkably, the frequency of methamphetamine dependence amongst female methadone dependent patients in the Iranian population was higher. This dependency has been linked to multiple health issues in the social and health contexts of both genders, particularly women [35].

Showing a possible association between *PICK1-rs713729* and *PICK1-rs2076369* in the *PICK1* gene promoter with methamphetamine dependence were the key findings of the present study. Consistent with our study, Matsuzawa et al*.* showed that *PICK1-rs713729* and *PICK1-rs2076369* were significantly associated with methamphetamine abuse in a Japanese population (Table 8). Additionally, they revealed that *PICK1-rs713729* was linked to those with spontaneous relapse of psychosis [16]. Moreover, *PICK1* gene was reported as the beginning of methamphetamine addiction, worse prognosis as well as spontaneous relapse [37]. *PICK1* is involved in the targeting and localization of synaptic membranes proteins and also in the surface of dopamine transporter clustering on the cell surface [18, 38]. This clustering leads to an increase of dopamine transporter uptake activity. Thus, *PICK1* expression levels can alter the role of the dopamine transporter and can affect the pathogenesis of methamphetamine abuse/dependency [16]. Moreover, the interactions of PICK1 with α-amino-3-hydroxy-5-methyl-4-isoxazole propionate (AMPA) receptors, metabotropic glutamate receptors and D-serine synthesizing enzyme have been shown and might be implicated in the pathogenesis of drug-related disorders [39–42]. Further studies are still required to show the direct association of these proteins in the pathogenesis of methamphetamine abuse.

**Table 8:** The association studies regarding methamphetamine and *BDNF*/*PICK1* gene polymorphisms in the methamphetamine abusers and controls

Gene-SNP	Ethnicity	Sample size		Result	Refs
		Meth	Normal		
BDNF-Val66Met	Chinese	200	219	No significant differences in genotype and allele. The Met allele was associated with earlier age onset of METH use	[52]
BDNF-rs16917204, rs16917234, rs2030324	Chinese	200	219	No significant differences in genotype and allele distributions	[53]
BDNF-Val66Met	Taiwanese	103	200	Significant differences in BDNF Val66Met genotype distribution	[21]
BDNF-Val66Met (rs6265)	Thai	100	102	Significant differences in the distribution of rs6265 genotype A lower frequency of GG genotype associated with METH-induced psychosis	[20]
BDNF promoter methylation	Chinese	30	52	BDNF promoter methylation is associated with drug addiction	[54]
BDNF-Val66Met	Chinese	194	378	No significant differences in genotype and allele distributions A significant positive correlation between serum BDNF and the delayed memory index score	[55]
BDNF-Val66Met	Japanese	189	202	No significant differences were found in the frequency of the genotype or allele	[56]
BDNF-Val66Met	Chinese	24	45	A significant distribution of the BDNF Val66Met genotype with METH dependence and METH psychosis in Chinese	[22]
	Malay	59	51		
	Kadazan-Dusun	50	30		
	Bajau	53	28		
	Total	186	154		
BDNF-Val66Met	Caucasian	60	None	Significantly more pretreatment days with METH use in females than males A significant association between females with Val/Val genotype and METH use	[57]
PICK1- s737622, rs3026682, rs713729, rs2076369	Japanese	208	218	A significantly association between: -rs713729 and METH abusers -rs713729 and rs2076369 with spontaneous relapse of psychosis	[16]

SNP: Single nucleotide polymorphism; PICK1: Protein that interact with C-kinase-1; BDNF: Brain-derived neurotrophic factor; Val66Met (also called rs6265): Met: Methionine and Val: Valine; METH: Methamphetamine; Ref: References

Based on previous studies, it was reported that gene variations, which are associated with glutamatergic and serotonin systems explain differences in SUD and/or dependency risk between individuals. It is indicated that glutamate-related genes influence the risk of SUD and/or dependency. The glutamate receptor genes were suggested to interact with BDNF by BDNF- tropomyosin kinase B (TrKB) transduction signaling cascade. BDNF is a neurotrophic factor, which is involved in the expansion, maintenance and survival of dopaminergic neurons in CNS [43].

In the present study, no association was found between *BDNF-rs6265* and the risk of methamphetamine dependence (SUD) in our population. This finding is consistent with the result of methamphetamine dependent male Caucasian individuals, which showed no association in 193 non-psychotic males (117 methamphetamine-dependent cases and 76 controls) [44]. However, Cheng et al*.* showed that there was an association between methamphetamine-dependency and *BDNF* gene in 103 methamphetamine abusers and 122 normal controls. They noted that the lower 66Met carriers were linked to substance abuse [21]. Furthermore, Sim et al*.* described an increase in a Chinese subgroup of Malaysian methamphetamine-dependent subjects (n = 24), which was not found among other Malaysian ethnic groups [22]. The various results for this SNP might be due to different sample sizes and also different ethnicities as well as genetic diversity (Table 8). It is indicated that ethnic differences can affect the frequency of BDNF Val66Met [45, 46].

Long-term methamphetamine-induced brain changes are significantly dependent on BDNF genetic variation [47]. Val66Met might enhance the risk of suicide behavior [45]. Moreover, BDNF variations might be involved in methamphetamine withdrawal. BDNF levels, equal or less than 1,251.0 pg/ml has been stated to be linked to depression symptoms during methamphetamine withdrawal [48]. BDNF Val66Met had an important effect on the Treatment Effectiveness Score (TES), methamphetamine-negative urine drug screens during treatment, which was higher among Val/Val Caucasian carriers [49]. Interestingly, there were more pretreatment days with methamphetamine use in females with Val/Val genotype than males [10]. Estrogen improves BDNF expression, which has implications on the release of striatal dopamine caused by methamphetamine and protects against neurotoxicity caused by methamphetamine [50, 51]. Thus, females can be able to use methamphetamine more frequently [10].

Furthermore, there was no association between *PICK1* and *BDNF* gene polymorphisms in our study. That may be attributed to the different positions of the gene polymorphisms on different chromosomes [*PICK1* (*22q13.1*) and *BDNF* (*11p13–15*)].

There were some limitations in our study. We did not investigate all the SNPs in these genes. Although, the sample size was large enough to detect an association between these SNPs and SUD in comparison with previous studies, larger sample size would strengthen the results. We therefore recommend further replication studies with larger sample size in order to validate and explain this association with conclusive findings.

## Conclusions

Collectively, the variation in the *PICK1* gene was associated with methamphetamine dependence (SUD), reflecting the underlying biological mechanisms, which can make a bridge between pathways and methamphetamine dependence (SUD). Our findings suggest that the *PICK1* gene might be involved in susceptibility to SUD in our population. These findings can be helpful in rehabilitation programs and psycho-education for those who have substance dependency. In this case, potential genetic predictors can be used for individuals susceptible to SUD. All in all, understanding genetic variations might help to understand the biological mechanisms of progression, and suppression of methamphetamine. In addition, our findings provide the basis for future genetic research on the use of methamphetamine dependency and related neurological side effects.

## Supplementary Information

**Additional file 1.** Haplotype frequencies in population.

**Additional file 2.** LD map and LD block.

## Data Availability

The datasets created during the current study are not publicly accessible due to the possibility of compromising the privacy of individuals. According to the written approval forms accepted by the Ethics Committee of the Mashhad University of Medical Sciences (MUMS), the data will only be available to researchers within project. The data would be available upon request from the corresponding authors (according to the MUMS rules and regulations).

## Abbreviations

AKT1: V-akt murine thymoma viral oncogene homolog 1
AMPA: α-Amino-3-hydroxy-5-methyl-4-isoxazole propionate
ARMS-PCR: Amplification refractory mutation system-polymerase chain reaction
ARRB2: Arrestin beta 2
BDNF: Brain-derived neurotrophic factor
CIs: Confidence intervals
CNS: Central nervous system
COMT: Catechol-O-methyltransferase
CYP2D6: Cytochrome P450 2D6
DRD4: Dopamine receptor D4
EDTA: Ethylene diamine tetra acetic acid
GABRA1: Gamma-aminobutyric acid type A receptor subunit alpha1
GABRG2: Gamma-aminobutyric acid receptor subunit gamma-2
GLYT1: Glycine transporter-1
GSTM1: Glutathione S-transferase mu 1
GSTP1: Glutathione S-transferase P1
HWE: Hardy–Weinberg equilibrium
LD: Linkage disequilibrium
MAF: Minor allele frequency
Nm: Nanometres
ORs: Odds ratios
PDYN: Prodynorphin
PICK1: Protein interacting with C kinase
SLC22A3: Solute carrier family 22 member 3
SNP: Single nucleotide polymorphism
SUD: Substance use disorder
TES: Treatment Effectiveness Score
TrKB: BDNF-tropomyosin kinase B
WASP: Web-based allele-specific primer designing tool
