# Supplementary material for: Association of PICK1 and BDNF variations with increased risk of methamphetamine dependence among Iranian population: a case–control study
Source: BMC Med Genomics. 2021 Jan 26;14:27. doi: 10.1186/s12920-021-00873-7 (PMC7836203; doi:10.1186/s12920-021-00873-7)
Supplement: Supplementary file 1 — Additional file 1. Haplotype frequencies in population. [file 12920_2021_873_MOESM1_ESM.docx]

**Additional file 1: Table 1.** Haplotype frequencies in population

| **Control samples** | | | |
| --- | --- | --- | --- |
| No | Haplotype_ID | Sequence | Frequency |
| 1 | H1 | TG | 0.57898 |
| 2 | H2 | TT | 0.34348 |
| 3 | H3 | AT | 0.04155 |
| 4 | H4 | AG | 0.03599 |
| **Case samples** | | | |
| No | Haplotype_ID | Sequence | Frequency |
| 1 | H1 | TG | 0.57417 |
| 2 | H2 | TT | 0.27492 |
| 3 | H3 | AG | 0.12853 |
| 4 | H4 | AT | 0.02237 |
| **Case & control samples** | | | |
| No | Haplotype_ID | Sequence | Frequency |
| 1 | H1 | TG | 0.57309 |
| 2 | H2 | TT | 0.30956 |
| 3 | H3 | AG | 0.08951 |
| 4 | H4 | AT | 0.02785 |

**H**: Haplotype; Haplotype frequencies for tested SNPs.
