# Supplementary material for: Association of PICK1 and BDNF variations with increased risk of methamphetamine dependence among Iranian population: a case–control study
Source: BMC Med Genomics. 2021 Jan 26;14:27. doi: 10.1186/s12920-021-00873-7 (PMC7836203; doi:10.1186/s12920-021-00873-7)
Supplement: Supplementary file 2 — Additional file 2. LD map and LD block. [file 12920_2021_873_MOESM2_ESM.docx]

**Additional file 2: Table 1.** LD map and LD block

| **Control samples** | | | | | | | |
| --- | --- | --- | --- | --- | --- | --- | --- |
| No | Marker1 | Marker2 | Distance | \|D'\| | r2 | LOD | P*__value_* |
| 1 | 0 | 1 | 8183 | 0.26 | 0.00915 | 1.629 | 0.06 |
| 2 | 0 | 2 | 10401093 | 0.43 | 0.00271 | 1.055 | 0.31 |
| 3 | 1 | 2 | 10409276 | 0.023 | 0.00014 | -0.019 | 0.82 |
| **Case samples** | | | | | | | |
| No | Marker1 | Marker2 | Distance | \|D'\| | r2 | LOD | P*__value_* |
| 1 | 0 | 1 | 8183 | 0.49 | 0.01879 | 6.88 | 0.003 |
| 2 | 0 | 2 | 10401093 | 0.07 | 0.00017 | 0.062 | 0.786 |
| 3 | 1 | 2 | 10409276 | 0.075 | 0.00244 | 0.719 | 0.29 |
| **Case & control samples** | | | | | | | |
| No | Marker1 | Marker2 | Distance | \|D'\| | r2 | LOD | P*__value_* |
| 1 | 0 | 1 | 8183 | 0.28 | 0.00540 | 3.379 | 0.03 |
| 2 | 0 | 2 | 10401093 | 0.17 | 0.00074 | 0.540 | 0.43 |
| 3 | 1 | 2 | 10409276 | 0.07 | 0.00184 | 0.287 | 0.21 |

**Marker1**: rs713729; **Marker2**: rs2076369; **Marker3**: rs6265; **LD:** Linkage disequilibrium; **LOD**: Logarithm of odds; * Significant of p< 0.05. Strong LD was defined as D^′^ ≥0.8.
